# Genomic insights into host–Endozoicomonadaceae cophylogeny

**DOI:** 10.1099/mgen.0.001384

**Published:** 2025-04-03

**Authors:** Zhuang Shao, Jian Zhang, Jiaxin Li, Jie Li

**Affiliations:** 1 CAS Key Laboratory of Tropical Marine Bio-Resources and Ecology, South China Sea Institute of Oceanology, Chinese Academy of Sciences, Guangzhou, 510301, PR China; 2 University of Chinese Academy of Sciences, Beijing, 100049, PR China

**Keywords:** comparative genomics, cophylogeny, cospeciation, *Endozoicomonadaceae*, host–bacteria interaction

## Abstract

The congruence between host and symbiont phylogenies reflects the evolutionary links among ecologically important interactions. As potential key symbionts, the members affiliated to the family *Endozoicomonadaceae* have previously been investigated for the cophylogenetic relationship with their hosts using their 16S rRNA gene sequences. However, this approach neglects the genomic features of symbionts that may influence the long-term associations between *Endozoicomonadaceae* members and their hosts. Here, we collected available high-quality genomes of *Endozoicomonadaceae* from diverse hosts and investigated their genomic features, including genome size, phages, insertion elements and the composition of functional genes. We also tested the host*–Endozoicomonadaceae* cophylogeny and examined the correlation between the cophylogenetic squared residuals and the genomic features of *Endozoicomonadaceae* members. Our results revealed a cophylogenetic pattern between members of the *Endozoicomonadaceae* family and their various hosts. Moreover, we found that the investigated genomes of *Endozoicomonadaceae* members were differentially eroded by phages and insertion elements. Additionally, *Endozoicomonadaceae* members with smaller, more eroded genomes tended to exhibit lower cophylogenetic residuals with their hosts. Gene function analysis further revealed that *Endozoicomonadaceae* members with closer associations with their hosts carried specific genes related to infection processes and host–symbiont interactions. This study suggests that the genomic features of *Endozoicomonadaceae* members may influence long-term host*–Endozoicomonadaceae* intimate associations.

**Impact Statement**

Given the wide distribution and ecological significance of members affiliated to the family *Endozoicomonadaceae*, it is essential to elucidate the relationship between hosts and *Endozoicomonadaceae* members from both ecological and phylogenetic perspectives. This study explored the potential correlation between the genomic features of the *Endozoicomonadaceae* family and long-term host*–Endozoicomonadaceae* coevolution. In general, our findings provide new insights into the evolution and ecological dynamics of hosts and symbionts

## Data Summary

All supporting data have been provided within the article or through supplementary data files.

## Introduction

The symbiotic relationship between microbes and marine animals is fundamental to the survival of marine organisms in various environments and underpins the health of marine ecosystems [1]. Among the diverse array of marine host-associated bacteria, the family *Endozoicomonadaceae* is notable for its abundance, commonness and functional diversity. Because of these features, it is regarded as a key group of bacterial symbionts in marine ecosystems [2,3]. The family *Endozoicomonadaceae* (*Gammaproteobacteria*, *Oceanospirillales*) [4] includes the genera *Kistimonas* [5], *Parendozoicomonas* [4], *Sansalvadorimonas* [6] and *Endozoicomonas* [7]. These bacteria inhabit a wide range of marine environments and various marine animals [8]. They are common bacterial symbionts of diverse invertebrates, such as stony corals, sponges, octocorals, sea anemones and sea squirts [9]. But there are also *Endozoicomonadaceae* species in seabream and deep-sea molluscs that were defined as pathogens [10,11]. Recent comparative genomic analyses revealed variability in the size of *Endozoicomonadaceae* genomes, which tend to be relatively large and exhibit varying degrees of erosion caused by phages and insertion elements [12,13]. Several studies have indicated that bacterial genome size can reflect the extent of its specialized symbiosis with the host [14,15]. Additionally, diverse insertion elements may help bacteria adapt to their hosts [16], potentially influencing gene functions or the evolution of bacteria [17,18]. Moreover, *Endozoicomonadaceae* species also harbour many genes related to symbiosis, such as the type III secretion system, cobalamin biosynthesis and dimethylsulfoniopropionate metabolism. These gene functions contribute to the host*–Endozoicomonadaceae* associations [12,16, 19]. Understanding the long-term, intimate relationships between symbionts and hosts is crucial for unravelling the ecological and evolutionary dynamics shaping host–symbiont interactions [20,21]. However, it remains uncertain to what extent the genomic features of *Endozoicomonadaceae* members reflect long-term associations with their hosts.

In this context, investigating the cophylogenetic pattern between members of *Endozoicomonadaceae* and their hosts becomes imperative. Understanding the evolutionary histories and coevolutionary dynamics between these symbiotic partners would provide insights into their interactions [22]. Previous studies have hinted at a cophylogenetic pattern between *Endozoicomonadaceae* members and their stony coral and sponge hosts based on 16S rRNA genes [23,24]. The limitations of using this marker gene may hinder a comprehensive understanding of the underlying mechanisms driving host–symbiont associations. Therefore, a genomic perspective is essential for elucidating the intricate relationships between *Endozoicomonadaceae* members and their hosts, especially given their ecological significance and wide distribution across marine ecosystems.

In this work, we collected nearly complete genomes of *Endozoicomonadaceae* members associated with marine invertebrates, fish, macroalgae and cold-seep environments, analysed the basic structure and functional features of these genomes and investigated the cophylogenetic pattern between *Endozoicomonadaceae* members and their hosts. We further explored the potential associations of *Endozoicomonadaceae* genome structure and gene functions with host*–Endozoicomonadaceae* cophylogenetic squared residuals. This study aims to investigate the genomic basis of host*–Endozoicomonadaceae* symbiosis and its implications for ecological and evolutionary dynamics.

## Methods

### *Endozoicomonadaceae* genome collections

The genomes (including cultivated genomes, single-cell amplified genomes and metagenomic assembled genomes) of the *Endozoicomonadaceae* members were collected from the National Center for Biotechnology Information (NCBI) and Zenodo (https://doi.org/10.5281/zenodo.7840163). These genomes were reannotated using Prokka v1.14.6 [25] to avoid the incongruence of different annotation schemes. They were checked for completeness, contamination, heterogeneity and other basic genome information with CheckM v1.0.12 [26]. Following the quality check, 42 *Endozoicomonadaceae* genomes were selected for this study. Thirty-eight of these genomes from shallow-sea hosts exhibited completeness ranging from 90 to 99.6% and contamination levels ranging from 0 to 5.2%. Additionally, four genomes from deep-sea invertebrate hosts and environmental samples showed completeness ranging from 73.8 to 88.1% and contamination levels ranging from 0 to 2.5%. Due to the rarity and difficulty of collecting these deep-sea samples, they were not filtered based on quality metrics (Table S1, available in the online Supplementary Material).

### Identification of genomic features

All orthologous groups of the genomes were identified using OrthoFinder v2.5.5 [27], and the visualization of orthologous groups in each genome was performed via UpSetR [28]. The whole-genome average nucleotide identity (ANI) was calculated using FastANI v1.32 [29]. Domain information, COG and KEGG orthology were annotated using eggNOG-mapper v2.1.12 [30]. Furthermore, EnrichM v0.6.5 (https://github.com/geronimp/enrichM) was used to reveal the complete KEGG orthology modules in the genomes. Enrichment analysis was used to map genes to pathways, and a network plot was generated to display shared genes between pathways, both using the clusterProfiler package [31]. The Phage Search Tool Enhanced Release [32] was used to detect the phage regions and identify the possible phage species. UGENE [33] was used to search for repeat sequences from genomes with a minimum identity of 98% and a minimum length threshold of 500 bp (rRNA was not included) [16]. The ISfinder [34] was used to further detect the insertion elements in the repeat sequences with the 10^−6^ e-value threshold [16]. The geNomad v1.7.4 [35] with the conservative filter was used to identify and annotate all phage sequences integrated into the genomes of *Endozoicomonadaceae* species. We performed two-tailed t-tests on the number of insertion elements and phages in the *Endozoicomonadaceae* genomes collected from stony corals and sponges. Similar t-tests were also conducted on *Endozoicomonadaceae* genomes collected from the families *Acroporidae* and *Pocilloporidae*. These groups were selected for t-tests based on the sufficient sample numbers required for the analysis.

### *Endozoicomonadaceae* and host phylogenetic reconstructions

Single-copy orthologous genes of *Endozoicomonadaceae* genomes were extracted from the OrthoFinder results. Each gene was aligned using muscle v5 [36], and low-quality alignments were removed using Gblocks v0.91b [37]. ModelFinder [38] was used to select the best evolutionary model. MrBayes v3.2 [39] was used for phylogenetic analysis with the WAG+G4 model, applying the Markov Chain Monte Carlo method using two independent runs of 2 000 000 generations, sampling at every generation with an initial burn-in of 1000 generations. *Hahella chejuensis* KCTC 2396 (*Oceanospirillales*, *Hahellaceae*) was selected as the outgroup. In the host phylogenetic analysis, hosts with clear species information were selected for phylogenetic reconstruction (except for the host *Diplodus puntazzo*, which was infected with *Endozoicomonadaceae* species under experimental conditions). *Tridacna crocea* was selected as the outgroup. For the selection of the host marker gene, representative sequences for 18S rRNA, cytochrome c oxidase subunit I and cytochrome b were collected from NCBI. These sequences were processed using the steps (muscle, Gblocks, ModelFinder and MrBayes with the GTR+G+F model) as those used for the phylogenetic reconstruction of *Endozoicomonadaceae* members.

### Cophylogenetic analysis

To test for phylogenetic congruence, the phylogenetic trees of the host and *Endozoicomonadaceae* were assessed using the Procrustean Approach to Cophylogeny (PACo) [40] with 100 000 permutations. This distance-based global fit method quantifies the topological congruence between the two phylogenetic trees. A *P*-value less than 0.01 indicates a strong, non-random association between the two phylogenies. Additionally, the squared residuals quantify the extent to which symbiont species track their hosts. A smaller squared residual indicates a closer cophylogenetic relationship between the host and symbiont. To improve the accuracy of the cophylogenetic analysis, additional host species of *Endozoicomonadaceae* were collected to identify other hosts in which these bacteria were found, beyond the ones from which they were originally isolated. We extracted 16S rRNA gene sequences from *Endozoicomonadaceae* genomes and performed a blastn analysis in NCBI. If the 16S rRNA gene sequences from *Endozoicomonadaceae* genomes exhibited more than 99% identity with other 16S gene sequences of *Endozoicomonadaceae* species in the database, we collected the corresponding host species information. A total of 22 *Endozoicomonadaceae* members with identified host species were subjected to cophylogenetic analysis (Table S1).

The software eMPRess [41] was used to assess the cophylogenetic events between *Endozoicomonadaceae* members and their hosts. Cophylogenetic events, including cospeciations, duplications, transfers and losses, were statistically tested through 100 generations of randomization, with a *P*-value lower than 0.01 indicating that the events were unlikely to have occurred by chance.

The cophylogenetic analysis was conducted at a bacterial family level, as higher taxonomic ranks are less likely to have evolved with their hosts, whilst lower taxonomic ranks may introduce more noise [23]. We also tested the correlation between the host*–Endozoicomonadaceae* cophylogenetic jackknifed squared residuals and the genome size, number of phages and total insertion elements using the Spearman method in RStudio [42].

## Results

### Genome features and phylogenomic analysis of the family *Endozoicomonadaceae*

**Fig. 1.**
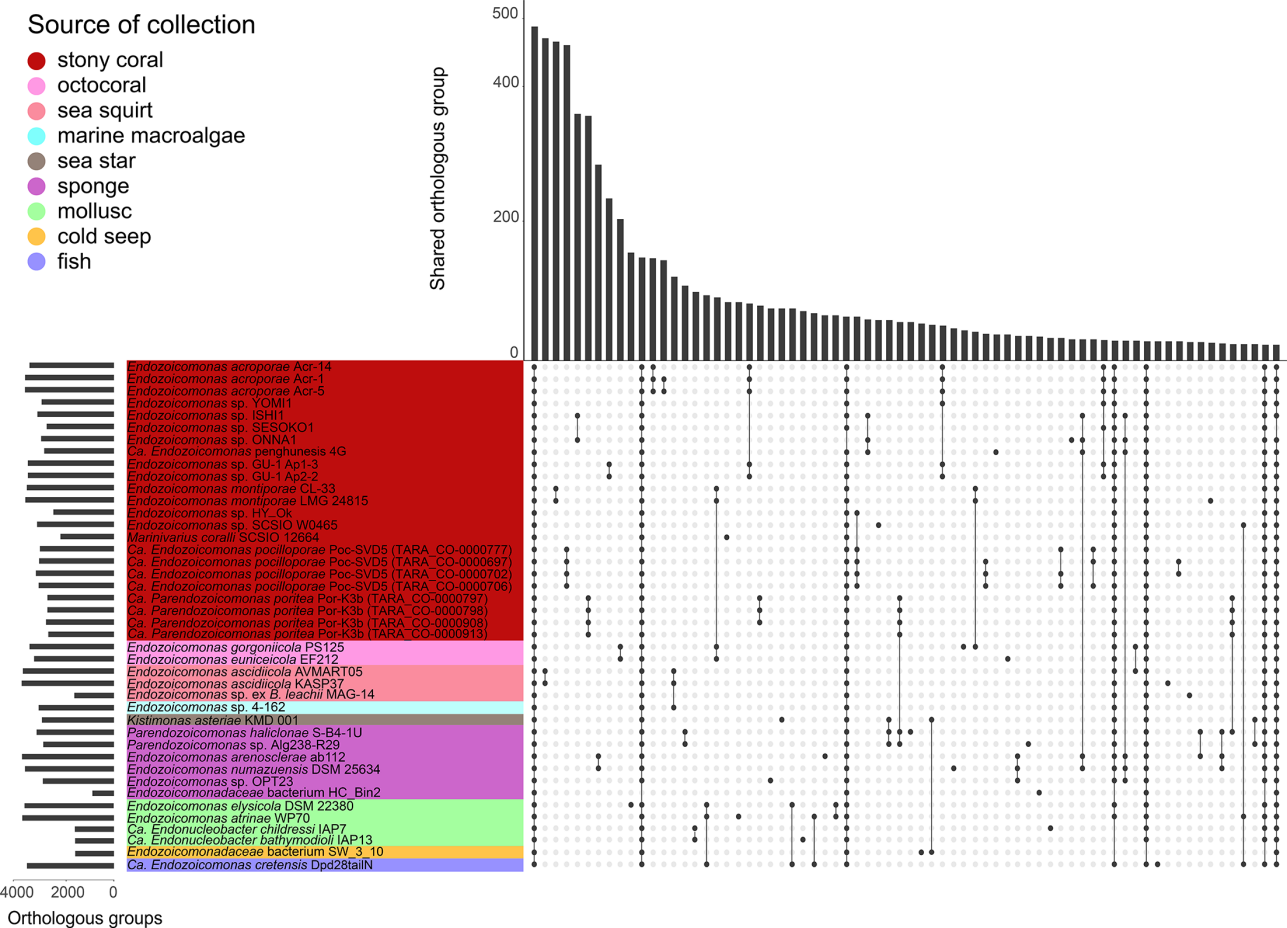
The number of orthologous groups in *Endozoicomonadaceae* genomes. The bar plot on the left indicates the number of orthologous groups in each genome. The bar plot on the top indicates the number of orthologous groups shared among the genomes. The dots and lines indicate the genomes from which the shared orthologous groups originate. The colours represent genomes collected from different sources.

This study encompassed a total of 42 genomes from *Endozoicomonadaceae* members, with genome sizes ranging from 2.00 to 6.98 Mb (calculated based on 100% completeness; Table S1). The largest genome, *Endozoicomonas* sp. SCSIO W0465, contained 6571 genes, whilst the smallest genome, *Endozoicomonadaceae* bacterium HC_Bin2, contained 1402 genes. In total, 190 375 genes were annotated across the 42 genomes. Approximately 2% of these genes were identified as single-copy orthologous groups shared by all genomes. A total of 94.9% of these genes were grouped into 14 567 orthologous clusters. Moreover, we found that two sea squirt-associated *Endozoicomonas ascidiicola* strains, AVMART05 and KASP37, shared 469 orthologous groups with a genome similarity of 98.77%. Similarly, the two stony coral-associated *Endozoicomonas montiporae* strains, CL-33 and LMG 24815, shared 464 orthologous groups with a genome similarity of 99.88% (Table S2). *Endozoicomonadaceae* members from each source of collection showed unique orthologous groups, except for uncultured *Endozoicomonas* sp. 4–162 collected from the marine macroalgae (Fig. 1).

**Fig. 2.**
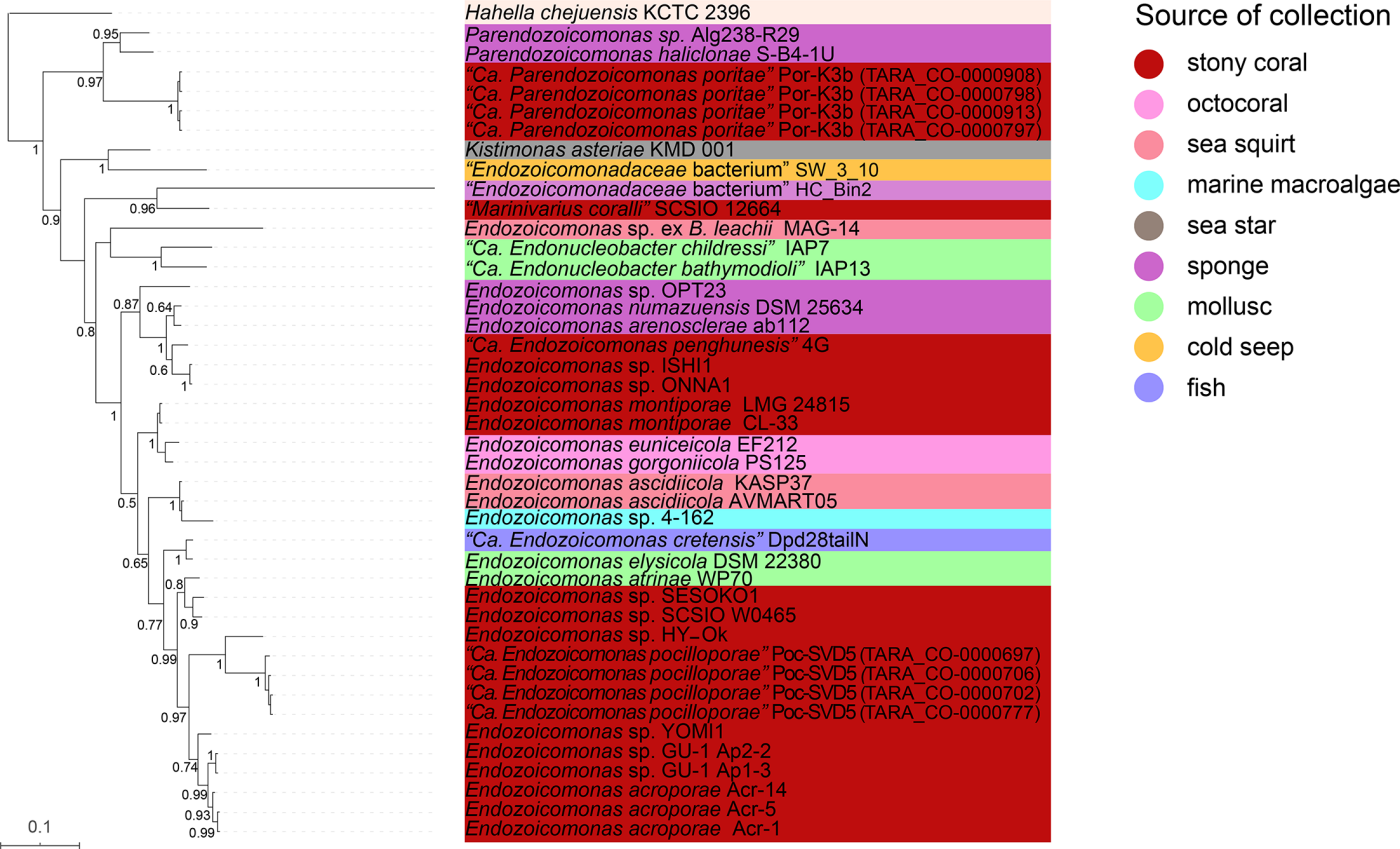
Phylogenomic analysis of the members of the *Endozoicomonadaceae* family. The evolutionary history was inferred using a Bayesian method based on 243 single-copy marker genes. The genome of *H. chejuensis* KCTC 2396 was used as the outgroup to root the tree. The coloured bars next to the tree display genus information of the genomes.

For the phylogenomic analysis, the phylogenetic tree of the members of *Endozoicomonadaceae* was constructed based on 243 single-copy marker genes. *Parendozoicomonas* members collected from the same type of host were observed to cluster together. Two unclassified strains, *Endozoicomonadaceae* bacterium SW_3_10 and *Endozoicomonadaceae* bacterium HC_Bin2, clustered separately with *Kistimonas asteriae* KMD 001 and ‘*Marinivarius coralli*’ SCSIO 12664, respectively. Most *Endozoicomonas* members collected from the same type of host were observed to cluster together, although those associated with stony corals formed three distinct groups (Fig. 2).

### Insertion elements in genomes of *Endozoicomonadaceae*

**Fig. 3.**
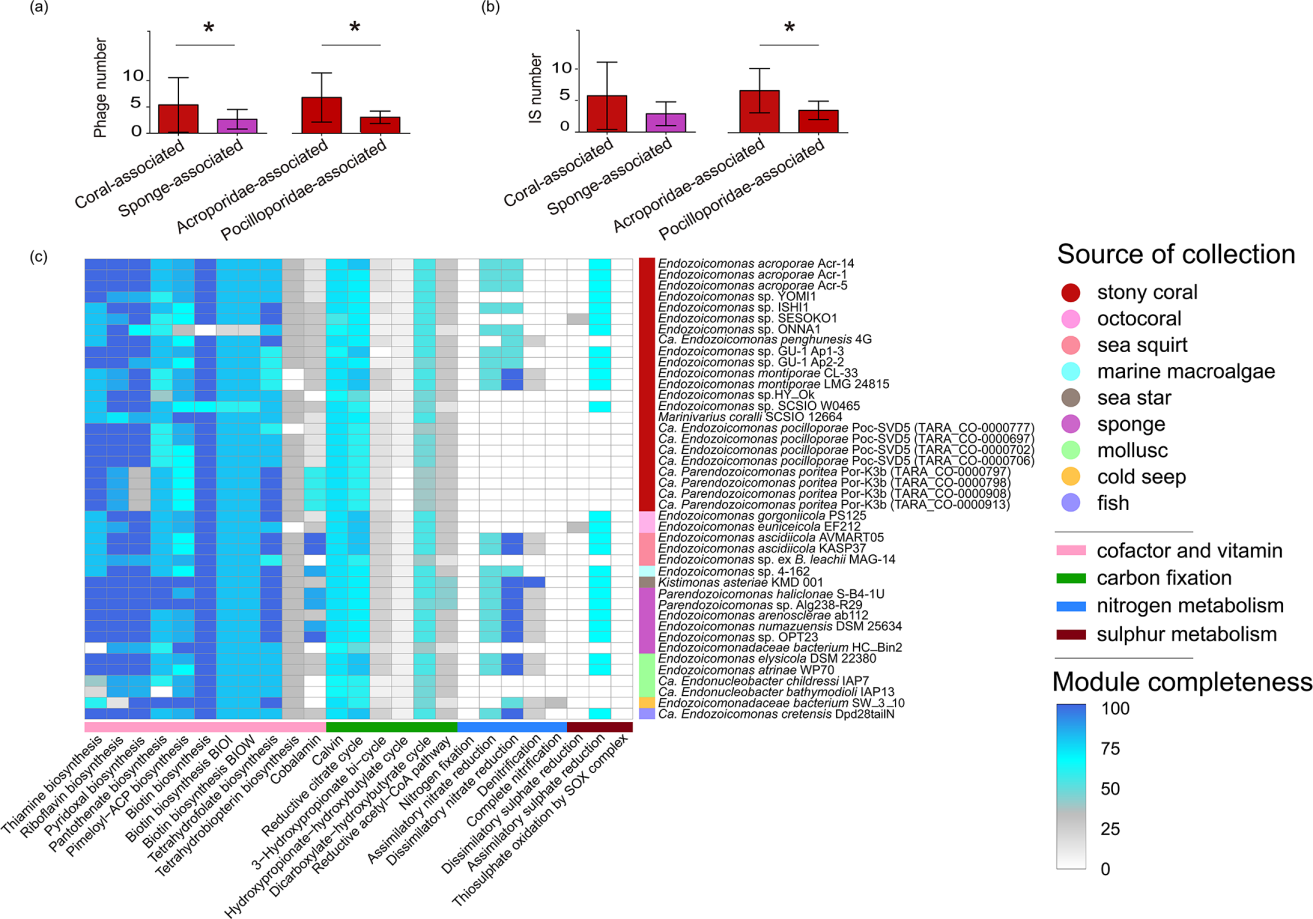
Differences in the number of phages and insertion elements, as well as in the metabolic features of *Endozoicomonadaceae* genomes. (a) T-tests comparing the number of phages in *Endozoicomonadaceae* genomes from different host sources. (b) T-tests comparing the number of insertion elements in *Endozoicomonadaceae* genomes from different host sources. (c) Major functional modules of *Endozoicomonadaceae* genomes. The colour gradient from white over grey to blue reflects an increase in the completeness of KEGG modules related to each function.

The number of insertion elements in *Endozoicomonadaceae* genomes ranged from 0 to 22, involving 13 distinct insertion element families (Table S1). No insertion elements were observed in *Endozoicomonadaceae* bacterium SW_3_10 collected from cold-seep seawater or in *Endozoicomonas* sp. OPT23 collected from *Ophlitaspongia papilla*, which may be attributed to their incomplete genomes (88 and 99% completeness, respectively). In the unpaired t-test with Welch’s correction (Fig. 3a and b), there was no significant difference in the number of insertion elements between stony coral-associated and sponge-associated *Endozoicomonadaceae* genomes (t=2.059, *P*=0.0505). However, a significant difference was observed in the number of insertion elements between *Endozoicomonadaceae* genomes associated with the families *Acroporidae* and *Pocilloporidae* (t=2.798, *P*=0.0151).

As a type of insertion element, the phage analysis revealed that a total of 2739 phage genes were annotated in all *Endozoicomonadaceae* genomes. Most phage species infecting *Endozoicomonadaceae* belong to the order *Caudoviricetes*, including *Autographiviridae*, *Ackermannviridae*, *Demerecviridae* and *Pedosvirus* (Table S3). By performing functional annotation of phage genes with Pfam, COG and KEGG databases, most of these genes encode phage proteins, such as phage major capsid protein E, phage tail tape-measure proteins and bacteriophage lambda head decoration protein D. However, we also found the vitamin B12 synthesis protein (cobalamin biosynthesis protein CobT) in phage sequences from *Endozoicomonas gorgoniicola* PS125 and *Endozoicomonas atrinae* WP70 (Table S3). Furthermore, different numbers of phages were observed between stony coral-associated and sponge-associated *Endozoicomonadaceae* genomes (t=2.102, *P*=0.046). A total of 106 phage regions were identified in the investigated *Endozoicomonadaceae* genomes collected from stony corals. Among these, 17 phages were intact, and PHAGE_Pseudo_phi3_NC_030940 was detected in eight genomes (Table S4). Additionally, a total of 14 phages were identified in the *Endozoicomonadaceae* genomes collected from sponges. Moreover, the number of phages was significantly different in the genomes of *Endozoicomonadaceae* members associated with *Acroporidae* and *Pocilloporidae* (t=2.658, *P*=0.0179).

### Metabolic potential of the *Endozoicomonadaceae* family

Through the annotation of gene functions in *Endozoicomonadaceae* genomes, eukaryotic repeat proteins (ankyrin repeats, WD40 repeats, tetratricopeptide repeat and leucine-rich repeat protein) were found in most (93%) of the studied *Endozoicomonadaceae* genomes. Genes of the type III secretion system were ubiquitous in all investigated *Endozoicomonadaceae* genomes (Table S5). The completeness of KEGG modules for putative gene functions of *Endozoicomonadaceae* genomes was assessed (Fig. 3c). In the cofactor and vitamin metabolism pathways, thiamine biosynthesis, riboflavin biosynthesis, pyridoxal biosynthesis and biotin biosynthesis were intact in most *Endozoicomonadaceae* genomes. *K. asteriae* KMD 001 and *Parendozoicomonas* sp. Alg238-R2 both exhibited seven complete cofactor and vitamin biosynthesis modules, except the pathways for tetrahydrobiopterin and cobalamin biosynthesis. ONNA1, associated with stony coral, only possessed the complete riboflavin biosynthesis module. *Endozoicomonas* sp. ex *B. leachii* MAG-14, *Ca. Endonucleobacter childressi* IAP7 and *Ca. Endonucleobacter bathymodioli* IAP13 retained the complete biotin biosynthesis module. *Endozoicomonadaceae* bacterium HC_Bin2, associated with sponge, lacked complete genes involved in thiamine, riboflavin, pyridoxal and tetrahydrofolate biosynthesis, whilst other sponge-associated members potentially produced these vitamins with complete biosynthetic modules. Additionally, results of functional gene analysis suggested that *Endozoicomonadaceae* did not appear to be capable of carbon fixation, nitrogen fixation, sulphate reduction or thiosulphate oxidation. Most coral-associated *Endozoicomonadaceae* members lacked complete genes involved in the assimilatory and dissimilatory nitrate reduction, denitrification and nitrification. *K. asteriae* KMD 001, associated with the sea star host, had the complete denitrification pathway. Additionally, the genes *nosZ*, *nosF* and *nosY*, responsible for the reduction of N_2_O, were observed in the genome of *K. asteriae* KMD 001. Phosphatidylcholine is the major membrane-forming phospholipid in eukaryotes and may be present in about 15% of bacteria [43]. Only *Endozoicomonas* sp. HY_Ok and *Ca. Endozoicomonas pocilloporae* Poc-SVD5 (TARA_CO-0000777) showed a complete phosphatidylcholine biosynthesis pathway. Ectoine acts as a cellular defence to protect cells against stress [44]. Amongst the *Endozoicomonadaceae* genomes, five contained a complete ectoine biosynthesis pathway, whilst the remaining genomes contained partial pathways (Table S6).

### Cophylogeny and correlation between cophylogenetic squared residuals and genomic features of the *Endozoicomonadaceae* family

**Fig. 4.**
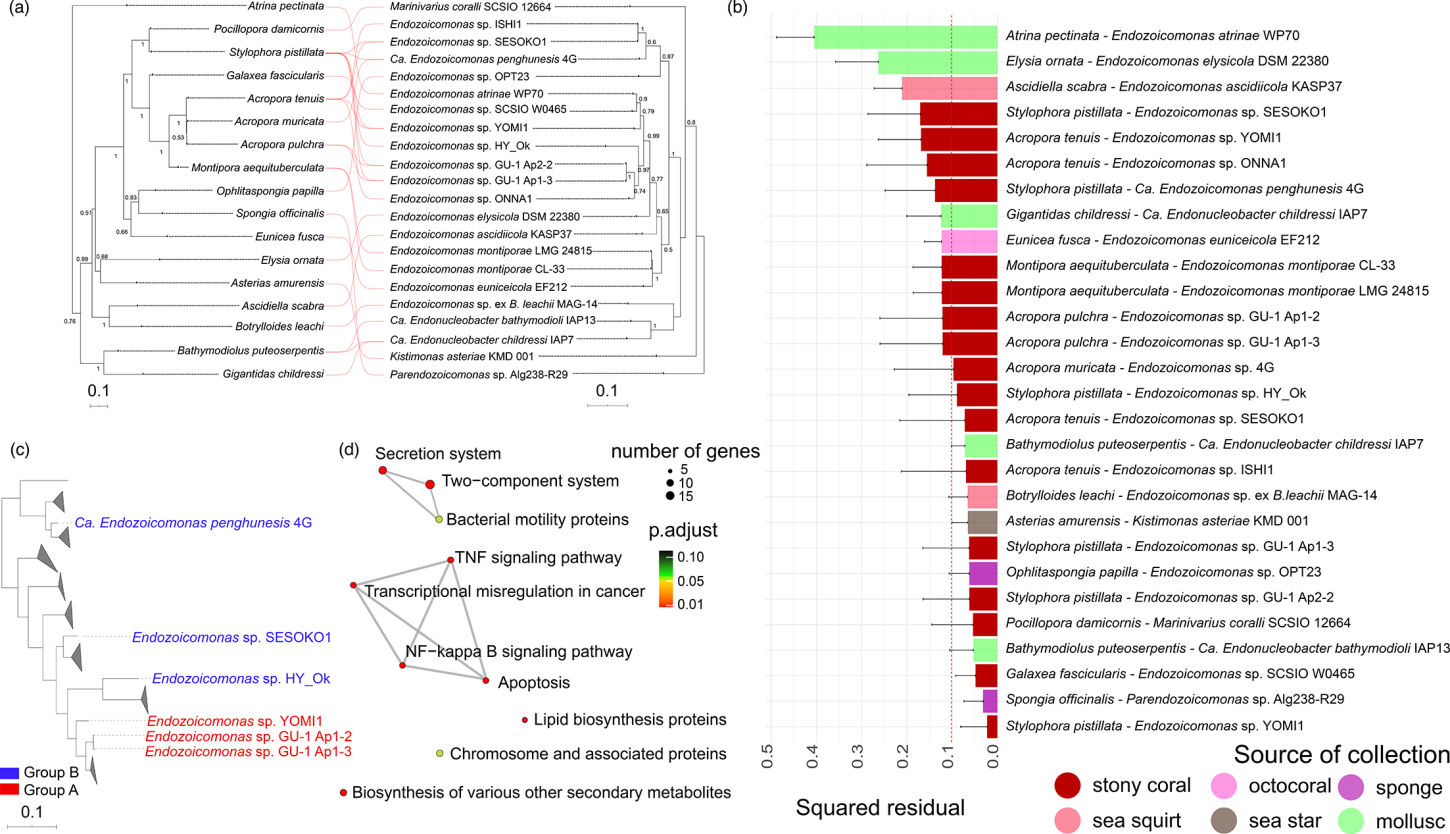
Cophylogenetic analysis of host*–Endozoicomonadaceae* and enrichment analysis of specific genes. (a) The tanglegram of host*–Endozoicomonadaceae*. (b) Contributions of individual host*–Endozoicomonadaceae* links to the procrustean fit. Jackknifed squared residuals (bars) and upper 95% CIs (error bars) resulting from applying PACo to the distances of *Endozoicomonadaceae* members. The colour of each bar indicates the source of collection. (c) The phylogeny of *Endozoicomonas* members. Members of *Endozoicomonas* collected from *S. pistillata* are categorized into groups A and B. (d) Enrichment analysis results of group A-specific genes for KEGG pathways. The top 10 pathways are displayed. The thickness of the lines among pathways indicates the number of shared genes. The sizes of the circles represent a scale, indicating the gene numbers in pathways.

Cophylogenetic analysis showed that the known members of the *Endozoicomonadaceae* family and their hosts had phylogenetic congruence (the observed m^2^ is 2.80251, *P*-value=4e-05<0.01), indicating that the phylogeny of *Endozoicomonadaceae* members was not randomly associated with the phylogeny of their hosts (Fig. 4a). In all 29 squared residuals, 15 residuals were lower than the median squared residual (Fig. 4b). The strongest cophylogenetic pattern (with the smallest squared residual) was observed between *Stylophora pistillata* and *Endozoicomonas* sp. YOMI1. The weakest cophylogenetic pattern was observed between *Atrina pectinata* and *E. atrinae*. The results of the Spearman correlation analysis suggested a potentially positive trend between the size of *Endozoicomonadaceae* genomes and the cophylogenetic squared residuals, although this trend did not reach statistical significance. Moreover, there appeared to be a negative trend between the number of insertion elements and phages in the genomes with the cophylogenetic squared residuals, though these relationships also lacked statistical significance (Fig. S1).

We further investigated the possible functional associations in the cophylogenetic process between *S. pistillata* and its associated *Endozoicomonas* symbionts on the basis of the relatively sufficient genomes. We divided the *Endozoicomonas* members collected from *S. pistillata* into two groups based on their cophylogenetic squared residuals: a smaller residual group A (*Endozoicomonas* sp. GU-1 Ap1-2, *Endozoicomonas* sp. GU-1 Ap1-3 and *Endozoicomonas* sp. YOMI1) and a larger residual group B (*Endozoicomonas* sp. HY_Ok, *Endozoicomonas* sp. SESOKO1 and *Ca. Endozoicomonas penghunesis* 4G) (Fig. 4b and c). In comparison of all the genes in group A with group B, we found that group A carried a total of 124 specific genes (Table S7). Enrichment analysis identified the top 10 pathways associated with these specific genes, involving the two-component system, secretion system, bacterial motility, lipid biosynthesis proteins, chromosome and associated proteins, biosynthesis of various other secondary metabolites and four pathways related to inflammation and immunity [45] (Fig. 4d).

The event-based cophylogenetic analysis using the eMPRess software revealed that host*–Endozoicomonadaceae* associations were involved in a total of 65 events, including 8 cospeciations, 9 duplications, 1 transfer and 47 loss events. The *P*-value from the randomization tests was less than 0.01 under cost region A (Fig. S2), indicating that the results were statistically significant and refuted the null hypothesis that the observed evolutionary events occurred by chance.

## Discussion

In this study, we gathered multiple *Endozoicomonadaceae* genomes along with their host information. The distance-based cophylogenetic analysis provided strong evidence for a cophylogenetic pattern between *Endozoicomonadaceae* members and their hosts. To further explore evolutionary events during the cophylogenetic process, we employed the event-based cophylogenetic analysis, which revealed potential cospeciation events between *Endozoicomonadaceae* members and their hosts. Moreover, as the host*–Endozoicomonadaceae* cophylogenetic squared residuals decreased, the genome size of *Endozoicomonadaceae* members tended to decrease, whilst the number of phages and insertion elements appeared to increase, although these relationships lacked statistical significance. Furthermore, by focusing on *S. pistillata*, a host harbouring a relatively large number of *Endozoicomonadaceae* members, we identified that *Endozoicomonadaceae* members with closer associations with their hosts carried specific genes associated with infection processes and host–symbiont interactions. This is the first study to investigate host*–Endozoicomonadaceae* cophylogeny from a genomic perspective.

Previous studies have shown that some *Endozoicomonadaceae* genomes carry a large number of symbiosis-related genes, including those involved in host adaptation and synthesis of B vitamins [3,46, 47], and our analysis supports this finding. A large number of type III secretion system genes were identified in all investigated *Endozoicomonadaceae* genomes, and 39 genomes contained eukaryotic repeat proteins (Table S5). Our analysis also revealed that *Endozoicomonadaceae* genomes exhibited significant potential in synthesizing B vitamins, especially B7 (biotin), B6 (pyridoxine), B2 (riboflavin) and B1 (thiamine). Additionally, we identified some genes in *Endozoicomonadaceae* genomes that may play a role in host*–Endozoicomonadaceae* adaptation. Ectoine, initially discovered as an osmoprotectant in anaerobic photobiology [48], was shown to enhance protein stability and act as a whole-cell stabilizer against various environmental stressors such as heating, freezing and UV radiation [44]. Among the genomes of the *Endozoicomonadaceae* family, five genomes had an intact pathway for ectoine biosynthesis, whilst others possessed a partially intact pathway (Table S6), suggesting that *Endozoicomonadaceae* members may exhibit the ability to adapt to complex microenvironments. A recent study [43] demonstrated that bacterial phosphatidylcholine plays an important role in the binding of bacteria to host macrophages and in promoting motility, biofilm formation and colonization of some pathogens, which may contribute to interactions between bacteria and their eukaryotic hosts. *Endozoicomonas* sp. HY_Ok and *Ca. E. pocilloporae* Poc-SVD5 (TARA_CO-0000777) had a complete phosphatidylcholine biosynthesis pathway (Table S6). However, the role of phosphatidylcholine in host*–Endozoicomonadaceae* interactions remains unknown. Moreover, our analysis of phages and insertion elements in *Endozoicomonadaceae* genomes revealed varying degrees of genome erosion (Table S1). The expression of phage genes during infection may confer new functions on bacteria [17,49]. We found that phages carrying the vitamin B12 synthesis protein (cobalamin biosynthesis protein CobT) were integrated into both *E. gorgoniicola* PS125 and *E. atrinae* WP70 (Table S3), suggesting that phage infections may facilitate functional associations between *Endozoicomonadaceae* members and their hosts.

Cophylogenetic patterns can reveal a prolonged evolutionary history of host–symbiont associations [50]. In our study, both distance-based and event-based analyses suggested that the evolutionary histories of the two pairwise comparisons (host*–Endozoicomonadaceae*) are not independent. The distance-based cophylogenetic analysis indicated significant phylogenetic congruence between the recognized *Endozoicomonadaceae* members and their hosts (Fig. 4a). Additionally, the notably small cophylogenetic squared residual observed between *S. pistillata* and *Endozoicomonas* sp. YOMI1 may underscore their close evolutionary association. The event-based cophylogenetic analysis identified an 8 out of 65 cospeciation rate, potentially contributing to the observed phylogenetic congruence. Moreover, we also found that the transfer event constituted only 1 out of 65 of all events, suggesting that members of the *Endozoicomonadaceae* family may rarely switch hosts. Additionally, the low transfer rate may also be attributed to the high cost of host transfer events, which are often replaced by duplicates and losses [51]. Whilst it is in our knowledge that a time-calibrated phylogeny may produce more accurate results when testing for cophylogenetic events [23,51], eMPRess, a recognized testing software, currently does not support this need. As a result, the timing of cophylogenetic events occurring between the members of the *Endozoicomonadaceae* family and their hosts cannot be accurately determined. Furthermore, when evaluating host–symbiont cophylogenetic events, the limited availability of symbiont data can result in incomplete phylogenetic branches for symbionts. This limitation may increase the occurrence of lost events [50] and reduce the proportion of evolutionary events favouring host–symbiont cophylogeny. A more comprehensive exploration of the host*–Endozoicomonadaceae* cophylogenetic relationship will require a more accurate and detailed analysis of a larger dataset.

The dependence of symbiont phylogeny on host phylogeny is a basic principle for testing cophylogeny [51], whilst the genome size of bacterial symbionts can reflect their evolutionary dependency on the host when they are engaged in obligate symbiosis, with smaller genomes indicating a more specialized symbiotic relationship [14]. Consistent with previous reports, the size of genomes varied widely among members of the *Endozoicomonadaceae* family investigated in this study, indicating the possibility of a facultative to obligate symbiotic life stage within the family of *Endozoicomonadaceae*. Moreover, a potential trend of decreased cophylogenetic squared residuals between *Endozoicomonadaceae* members and their hosts as the *Endozoicomonadaceae* genomes shrank, although this trend was not statistically significant (Fig. S1A), suggesting that *Endozoicomonadaceae* genome size may reflect the host*–Endozoicomonadaceae* cophylogenetic relationship, providing a new perspective for understanding the host–symbiont cophylogeny.

In addition to genome size, a variety of phages and insertion elements offer insights into the infection and colonization histories of various marine hosts, facilitated by frequent divergence occurrences [12]. Furthermore, we observed potential trends showing increased cophylogenetic squared residuals between *Endozoicomonadaceae* species and their hosts as the number of phages and insertion elements increased, though these relationships were not statistically significant (Fig. S1B and C). Infection of bacterial genomes by phages and insertion elements causes alterations in genome structure and promotes the horizontal transfer of genes [52,53]. This process contributes to bacterial adaptation and evolution [54] and may also play a role in host–bacteria cophylogeny.

Bacteria that exhibit a cophylogenetic pattern with their hosts may be functionally interdependent with them, fostering stable symbiotic relationships over long evolutionary periods [55,56]. In our analysis, *Endozoicomonadaceae* members in group A, which had stronger connections with the host *S. pistillata* than those in group B, carried specific genes related to the two-component system, bacterial motility proteins and immune response (Fig. 4d). These functions may play a role in processes including host infection and interaction [45,57, 58], contributing to the long-term symbiosis between hosts and *Endozoicomonadaceae* members. Therefore, grouping symbionts based on their phylogenetic positions and the cophylogenetic squared residuals of host–symbiont pairs may be an effective method for elucidating the long-term functional associations between hosts and symbionts.

In contrast to previous analyses of host*–Endozoicomonadaceae* cophylogeny based on *Endozoicomonadaceae* 16S rRNA genes [23,24], our study firstly demonstrates the cophylogenetic pattern between *Endozoicomonadaceae* members and various marine hosts based on *Endozoicomonadaceae* genomes. Secondly, this study discusses the potential correlation between the genomic features of investigated *Endozoicomonadaceae* members, such as genome size, phages, insertion elements and gene functions, and their long-term symbiotic relationship with their hosts. We suggest that the genomic features of symbionts should be considered in future studies of host–symbiont cophylogeny.

## supplementary material

10.1099/mgen.0.001384 Uncited Supplementary Material 1.

10.1099/mgen.0.001384 Uncited Supplementary Material 2.

## Abbreviations

ANI: average nucleotide identity
NCBI: National Center for Biotechnology Information
PACo: Procrustean Approach to Cophylogeny

## References

[1] Wilkins LG, Leray M, O’Dea A, Yuen B, Peixoto RS (2019). Host-associated microbiomes drive structure and function of marine ecosystems. PLoS Biol.

[2] Neave MJ, Apprill A, Ferrier-Pagès C, Voolstra CR (2016). Diversity and function of prevalent symbiotic marine bacteria in the genus *Endozoicomonas*. Appl Microbiol Biotechnol.

[3] Hochart C, Paoli L, Ruscheweyh H-J, Salazar G, Boissin E (2023). Ecology of *Endozoicomonadaceae* in three coral genera across the Pacific Ocean. Nat Commun.

[4] Bartz J-O, Blom J, Busse H-J, Mvie JB, Hardt M (2018). *Parendozoicomonas haliclonae* gen. nov. sp. nov. isolated from a marine sponge of the genus *Haliclona* and description of the family *Endozoicomonadaceae fam*. nov. comprising the genera *Endozoicomonas*, *Parendozoicomonas*, and *Kistimonas*. Syst Appl Microbiol.

[5] Choi EJ, Kwon HC, Sohn YC, Yang HO (2010). *Kistimonas asteriae* gen. nov., sp. nov., a gammaproteobacterium isolated from *Asterias amurensis*. Int J Syst Evol Microbiol.

[6] Goldberg SR, Haltli BA, Correa H, Kerr RG (2018). Description of *Sansalvadorimonas verongulae* gen. nov., sp. nov., a gammaproteobacterium isolated from the marine sponge *Verongula gigantea*. Int J Syst Evol Microbiol.

[7] Kurahashi M, Yokota A (2007). *Endozoicomonas elysicola* gen. nov., sp. nov., a gamma-proteobacterium isolated from the sea slug *Elysia ornata*. Syst Appl Microbiol.

[8] da Silva DMG, Pedrosa FR, Ângela Taipa M, Costa R, Keller-Costa T (2023). Widespread occurrence of chitinase-encoding genes suggests the *Endozoicomonadaceae* family as a key player in chitin processing in the marine benthos. ISME Commun.

[9] Shiu J-H, Tang S-L (2019). Symbiotic Microbiomes of Coral Reefs Sponges and Corals.

[10] Qi W, Cascarano MC, Schlapbach R, Katharios P, Vaughan L (2018). *Ca. Endozoicomonas cretensis*: a novel fish pathogen characterized by genome plasticity. Genome Biol.

[11] González Porras MÁ, Assié A, Tietjen M, Violette M, Kleiner M (2011). An intranuclear bacterial parasite of deep-sea mussels expresses apoptosis inhibitors acquired from its host. bioRxiv.

[12] Tandon K, Lu C-Y, Chiang P-W, Wada N, Yang S-H (2020). Comparative genomics: dominant coral-bacterium *Endozoicomonas acroporae* metabolizes dimethylsulfoniopropionate (DMSP). ISME J.

[13] Neave MJ, Michell CT, Apprill A, Voolstra CR (2017). *Endozoicomonas genomes* reveal functional adaptation and plasticity in bacterial strains symbiotically associated with diverse marine hosts. Sci Rep.

[14] Moran NA, McCutcheon JP, Nakabachi A (2008). Genomics and evolution of heritable bacterial symbionts. Annu Rev Genet.

[15] McCutcheon JP, Moran NA (2012). Extreme genome reduction in symbiotic bacteria. Nat Rev Microbiol.

[16] Ding J-Y, Shiu J-H, Chen W-M, Chiang Y-R, Tang S-L (2016). Genomic insight into the host*-Endosymbiont relationship* of *Endozoicomonas montiporae* CL-33(T) with its coral host. Front Microbiol.

[17] Chevallereau A, Pons BJ, van Houte S, Westra ER (2022). Interactions between bacterial and phage communities in natural environments. Nat Rev Microbiol.

[18] Navarro F, Muniesa M (2017). Phages in the human body. Front Microbiol.

[19] Pogoreutz C, Oakley CA, Rädecker N, Cárdenas A, Perna G (2022). Coral holobiont cues prime *Endozoicomonas* for a symbiotic lifestyle. ISME J.

[20] Gupta A, Nair S (2020). Dynamics of insect-microbiome interaction influence host and microbial symbiont. Front Microbiol.

[21] Brinker P, Fontaine MC, Beukeboom LW, Salles JF (2019). Host, symbionts, and the microbiome: the missing tripartite interaction. Trends Microbiol.

[22] Johnston EC, Cunning R, Burgess SC (2022). Cophylogeny and specificity between cryptic coral species (*Pocillopora* spp.) at Mo’orea and their symbionts (Symbiodiniaceae). Mol Ecol.

[23] Pollock FJ, McMinds R, Smith S, Bourne DG, Willis BL (2018). Coral-associated bacteria demonstrate phylosymbiosis and cophylogeny. Nat Commun.

[24] O’Brien PA, Andreakis N, Tan S, Miller DJ, Webster NS (2021). Testing cophylogeny between coral reef invertebrates and their bacterial and archaeal symbionts. Mol Ecol.

[25] Seemann T (2014). Prokka: rapid prokaryotic genome annotation. Bioinform.

[26] Parks DH, Imelfort M, Skennerton CT, Hugenholtz P, Tyson GW (2015). CheckM: assessing the quality of microbial genomes recovered from isolates, single cells, and metagenomes. Genome Res.

[27] Emms DM, Kelly S (2018). OrthoFinder2: fast and accurate phylogenomic orthology analysis from gene sequences. BioRxiv.

[28] Conway JR, Lex A, Gehlenborg N (2017). UpSetR: an R package for the visualization of intersecting sets and their properties. Bioinform.

[29] Jain C, Rodriguez-R LM, Phillippy AM, Konstantinidis KT, Aluru S (2018). High throughput ANI analysis of 90K prokaryotic genomes reveals clear species boundaries. Nat Commun.

[30] Cantalapiedra CP, Hernández-Plaza A, Letunic I, Bork P, Huerta-Cepas J (2021). eggNOG-mapper v2: functional annotation, orthology assignments, and domain prediction at the metagenomic scale. Mol Biol Evol.

[31] Yu G, Wang L-G, Han Y, He Q-Y (2012). clusterProfiler: an R package for comparing biological themes among gene clusters. OMICS.

[32] Arndt D, Grant JR, Marcu A, Sajed T, Pon A (2016). PHASTER: a better, faster version of the PHAST phage search tool. Nucleic Acids Res.

[33] Okonechnikov K, Golosova O, Fursov M, Team U (2012). Unipro UGENE: a unified bioinformatics toolkit. Bioinform.

[34] Siguier P, Perochon J, Lestrade L, Mahillon J, Chandler M (2006). ISfinder: the reference centre for bacterial insertion sequences. Nucleic Acids Res.

[35] Camargo AP, Roux S, Schulz F, Babinski M, Xu Y (2005). You can move, but you can’t hide: identification of mobile genetic elements with geNomad. Bioinform.

[36] Edgar RC (2020). MUSCLE v5 enables improved estimates of phylogenetic tree confidence by ensemble bootstrapping. BioRxiv.

[37] Talavera G, Castresana J (2007). Improvement of phylogenies after removing divergent and ambiguously aligned blocks from protein sequence alignments. Syst Biol.

[38] Kalyaanamoorthy S, Minh BQ, Wong TK, von Haeseler A, Jermiin LS (2017). ModelFinder: fast model selection for accurate phylogenetic estimates. Nat Methods.

[39] Ronquist F, Teslenko M, van der Mark P, Ayres DL, Darling A (2012). MrBayes 3.2: efficient bayesian phylogenetic inference and model choice across a large model space. Syst Biol.

[40] Hutchinson MC, Cagua EF, Balbuena JA, Stouffer DB, Poisot T (2017). paco: implementing procrustean approach to cophylogeny in R. Methods Ecol Evol.

[41] Santichaivekin S, Yang Q, Liu J, Mawhorter R, Jiang J (2021). eMPRess: a systematic cophylogeny reconciliation tool. Bioinform.

[42] Racine JS (2012). RStudio: A Platform-Independent IDE for R and Sweave.

[43] Geiger O, López-Lara IM, Sohlenkamp C (2013). Phosphatidylcholine biosynthesis and function in bacteria. Biochim Biophys Acta, Mol Cell Biol Lipids.

[44] Chen J, Qiao D, Yuan T, Feng Y, Zhang P (2024). Biotechnological production of ectoine: current status and prospects. Folia Microbiol.

[45] Ide K, Nishikawa Y, Maruyama T, Tsukada Y, Kogawa M (2022). Targeted single-cell genomics reveals novel host adaptation strategies of the symbiotic bacteria *Endozoicomonas* in *Acropora tenuis* coral. Microbiome.

[46] Alex A, Antunes A (2019). Comparative genomics reveals metabolic specificity of *Endozoicomonas* isolated from a marine sponge and the genomic repertoire for host-bacteria symbioses. Microorganisms.

[47] Keller-Costa T, Kozma L, Silva SG, Toscan R, Gonçalves J (2022). Metagenomics-resolved genomics provides novel insights into chitin turnover, metabolic specialization, and niche partitioning in the octocoral microbiome. Microbiome.

[48] Galinski EA, Pfeiffer HP, Trüper HG (1985). 1,4,5,6-tetrahydro-2-methyl-4-pyrimidinecarboxylic acid. a novel cyclic amino acid from halophilic phototrophic bacteria of the genus *Ectothiorhodospira*. Eur J Biochem.

[49] Zhou K, Zhang T, Chen X-W, Xu Y, Zhang R (2024). Viruses in marine invertebrate holobionts: complex interactions between phages and bacterial symbionts. Ann Rev Mar Sci.

[50] Groussin M, Mazel F, Alm EJ (2020). Co-evolution and co-speciation of host-gut bacteria systems. Cell Host Microbe.

[51] de Vienne D, Refrégier G, López-Villavicencio M, Tellier A, Hood M (2013). Cospeciation vs host-shift speciation: methods for testing, evidence from natural associations and relation to coevolution. New Phytol.

[52] Bennett PM (2004). Genomics, Proteomics, and Clinical Bacteriology: Methods and Reviews.

[53] Siguier P, Gourbeyre E, Chandler M (2014). Bacterial insertion sequences: their genomic impact and diversity. FEMS Microbiol Rev.

[54] Durrant MG, Li MM, Siranosian BA, Montgomery SB, Bhatt AS (2020). A Bioinformatic Analysis of Integrative Mobile Genetic Elements Highlights Their Role in Bacterial Adaptation. Cell Host Microbe.

[55] Janzen DH (1980). When is it coevolution. Evolution.

[56] Thompson JN (1994). The Coevolutionary Process.

[57] Raina J-B, Fernandez V, Lambert B, Stocker R, Seymour JR (2019). The role of microbial motility and chemotaxis in symbiosis. Nat Rev Microbiol.

[58] Bélanger L, Dimmick KA, Fleming JS, Charles TC (2009). Null mutations in *Sinorhizobium meliloti* exoS and chvI demonstrate the importance of this two-component regulatory system for symbiosis. Mol Microbiol.

