# Supplementary material for: Genomic insights into host–Endozoicomonadaceae cophylogeny
Source: Microb Genom. 2025 Apr 3;11(4):001384. doi: 10.1099/mgen.0.001384 (PMC11968832; doi:10.1099/mgen.0.001384)
Supplement: Uncited Supplementary Material 1. [file mgen-11-01384-s001.pdf]

Supplementary Figures for

Genomic insights into host-*Endozoicomonadaceae* cophylogeny

Author names

Zhuang Shao[a, b], Jian Zhang[a], Jiaxin Li[a, b], Jie Li[a, *]

Affiliation(s)

[a]CAS Key Laboratory of Tropical Marine Bio-resources and Ecology, South China Sea Institute of Oceanology, Chinese Academy of Sciences, Guangzhou, 510301, China

[b]University of Chinese Academy of Sciences, Beijing, 100049, China

Corresponding author and email address

Jie Li, lijietaren@scsio.ac.cn

Supplementary Figures

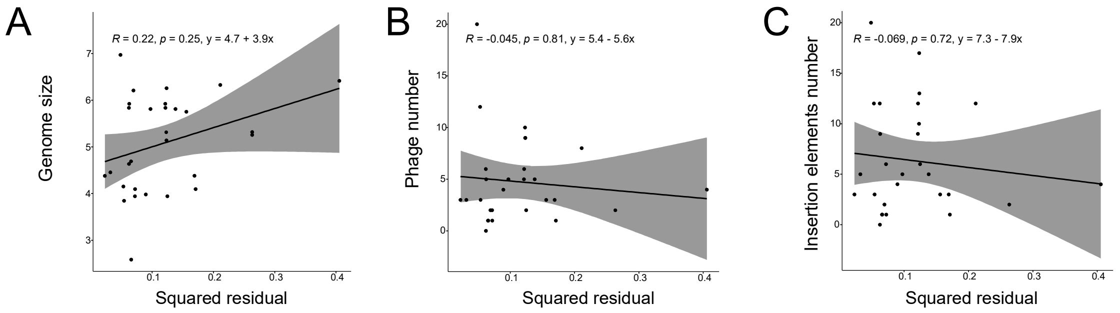

Fig. S1. The Spearman correlation of genome features with cophylogenetic squared residuals. A. The Spearman correlation between genome size and cophylogenetic squared residuals. B. The Spearman correlation between the number of phages and cophylogenetic squared residuals. C. The Spearman correlation between the number of insertion elements and cophylogenetic squared residuals. The gray shaded areas represent the 95% confidence intervals, and the straight lines represent the results of the linear regression.

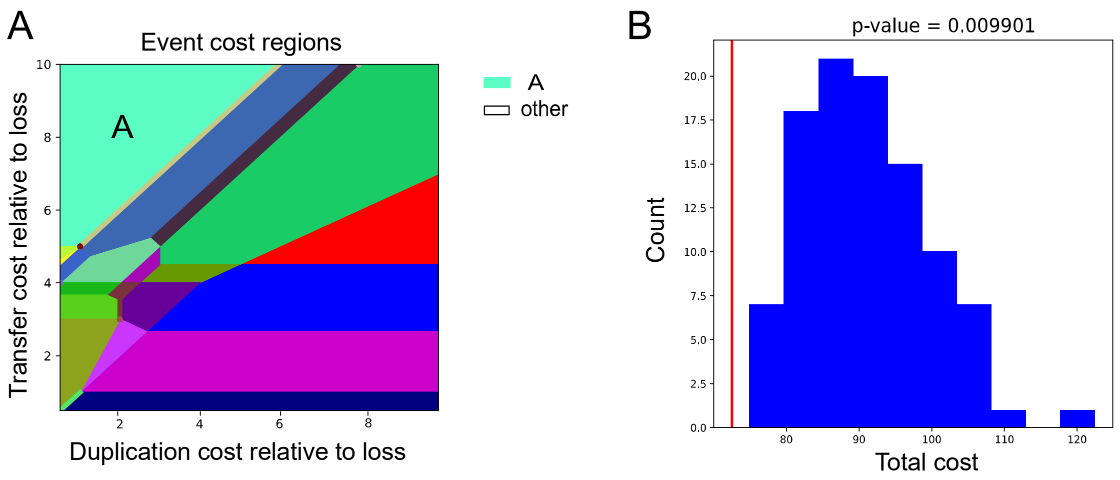

Fig. S2. Cophylogenetic event cost regions and significance of maximum parsimony reconciliation. A. Maximum parsimony reconciliation proposed for the cost region A (top left) and other regions. B. P-value histogram of maximum parsimony reconciliation under region A.
